# Draft *Anaplasma phagocytophilum* Genome Sequences from Five Cows, Two Horses, and One Roe Deer Collected in Europe

**DOI:** 10.1128/genomeA.00950-16

**Published:** 2016-12-01

**Authors:** Thibaud Dugat, Marie-Noëlle Rossignol, Olivier Rué, Valentin Loux, Sylvain Marthey, Marco Moroldo, Cornelia Silaghi, Dirk Höper, Julia Fröhlich, Martin Pfeffer, Erich Zweygarth, Anne-Claire Lagrée, Henri-Jean Boulouis, Nadia Haddad

**Affiliations:** a Université Paris-Est, Agence nationale de sécurité sanitaire de l’alimentation, de l’environnement et du travail, Laboratoire de santé animale, UMR BIPAR, Maisons-Alfort, France; b GABI, INRA, AgroParisTech, Université Paris-Saclay, Jouy-en-Josas, France; c MaIAGE, INRA, Université Paris-Saclay, Jouy-en-Josas, France; d Comparative Tropical Medicine and Parasitology, Ludwig-Maximilians-Universität München, Munich, Germany; e Institute of Diagnostic Virology, Friedrich-Loeffler-Institut, Federal Research Institute for Animal Health, Insel Riems, Germany; f Université Paris-Est, École Nationale Vétérinaire d’Alfort, UMR BIPAR ENVA Anses UPEC USC INRA, Maisons-Alfort, France

## Abstract

*Anaplasma phagocytophilum* is a zoonotic tick-borne intracellular bacterium responsible for granulocytic anaplasmosis. As it is difficult to isolate and cultivate, only 20 *A. phagocytophilum* genomes have been sequenced to date. Here, we present eight *A. phagocytophilum* genome sequences obtained using alternative approaches based on sequence capture technology.

## GENOME ANNOUNCEMENT

*Anaplasma phagocytophilum* is a zoonotic and obligate intracellular bacterium transmitted by hard ticks. It is the causative agent of tick-borne fever in ruminants, a disease which causes significant economic losses in Europe, and of equine granulocytic anaplasmosis (EGA) in horses, of canine granulocytic anaplasmosis (CGA) in dogs, and of human granulocytic anaplasmosis (HGA) in both the United States and Europe (1). *A. phagocytophilum* is difficult to isolate and cultivate, and thus only 20 genomes have been sequenced to date, among which only four originate from European samples. In particular, there are no *A. phagocytophilum* genomes from horse or roe deer samples, even though EGA can have such important economic impact (2), and roe deer are suspected to play a crucial role as reservoir hosts in *A. phagocytophilum* epidemiology (1). Here, we present the draft genome sequences of European *A. phagocytophilum* from four cow and two horse samples, and two strains isolated from one cow and one roe deer which had been maintained in continuous cell cultures (3, 4).

To overcome limitations due to the lack of reliable, easy, and feasible protocols to isolate *A. phagocytophilum*, we decided to follow a different strategy in order to obtain these genome sequences. We used a whole-genome sequence capture approach, whose efficacy on *A. phagocytophilum* has already been demonstrated (5). For each sample, total genomic DNA was extracted from whole blood of the animal, or from bacterial culture in IDE8 tick cell lines, using the NucleoSpin Blood QuickPure kit (Macherey-Nagel, Bethlehem, USA). The six bacterial genomes from animal blood were then captured and sequenced as previously described (5), but in this case a liquid-phase protocol based on the SeqCap EZ library kit (NimbleGen, Madison, USA) was used instead of solid-phase. All eight samples were sequenced on a single flow cell lane of a HiSeq3000 (Illumina, San Diego, USA) sequencer as paired-end 150 bp reads (insert-size: 280 ± 50 bp).

First, overlapping paired-end reads were merged with Flash (6) and were trimmed with Sickle (https://github.com/najoshi/sickle) (−n −q 24 −l 100). Reads were digitally normalized using Khmer (7) with a k-mer size of 20 and a cutoff of 20. Then, the remaining reads were mapped to the host genomes [*Bos taurus* (UMD3.1*), Equus caballus* (EquCab2.0), and *Capreolus capreolus* (CCMK000000000)] and to *A. phagocytophilum* HZ (NC_007797.1), using the BWA algorithm (v0.6.1) with default parameters (8). Only those reads mapping to *A. phagocytophilum* or which remained unmapped were retained. Three sets of reads were used for assembly processing: paired-end reads mapped on *A. phagocytophilum*, paired-end unmapped reads, and singleton reads (unmapped or mapped to *A. phagocytophilum*). Those reads were then assembled using Spades (9), with a k-mer value from 81 to 125 with a step of 4, and default parameters. Genome annotation was performed using Prokka. Genome characteristics are summarized in Table 1.

**TABLE 1:** Genome sequence accession numbers

Sample	Nucleotide sequence accession no.	Host	Geographical origin	Genome size (bp)	No. of contigs (scaffolds)	G+C (%)	No. of genes
Cow_1	FLLR01000001–FLLR01000249	Cow	France	1,682,137	249	41.93	1,519
Cow_2	FLMA01000001–FLMA01000230	Cow	France	1,641,350	230	42.23	1,463
Cow_3	FLMB01000001–FLMB01000191	Cow	France	1,561,654	191	42.00	1,417
Cow_4	FLLZ01000001–FLLZ01000230	Cow	France	1,604,419	230	42.12	1,448
Cow_5	FLMD02000001–FLMD02000300	Cow	Germany	2,196,284	300	43.70	1,979
Horse_1	FLMF02000001–FLMF02000300	Horse	France	1,784,294	300	41.90	1,657
Horse_2	FLMC02000001–FLMC02000300	Horse	France	2,191,611	300	41.58	1,997
Roe_deer_1	FLME02000001–FLME02000300	Roe deer	Germany	2,120,290	300	42.72	1,912

### Accession number(s).

The eight draft genomes have been deposited in the European Nucleotide Archive (Table 1).
